# Symptoms of gastroesophageal reflux disease in severely mentally retarded people: a systematic review

**DOI:** 10.1186/1471-230X-8-23

**Published:** 2008-06-11

**Authors:** Anke JE de Veer, Judith T Bos, Riet C Niezen-de Boer, Clarisse JM Böhmer, Anneke L Francke

**Affiliations:** 1 Netherlands institute for health services research, Utrecht, The Netherlands; 2 Bartiméus, Doorn, The Netherlands; 3 Spaarne Hospital, Hoofddorp, The Netherlands; 4 NIVEL, P.O. Box 1568, 3500 BN Utrecht, The Netherlands

## Abstract

**Background:**

Gastroesophageal reflux disease (GERD) occurs when stomach acid frequently backs up (or refluxes) into the gullet (or esophagus), and it has serious consequences for the quality of life. Usually this is felt as heartburn. Because severely mentally retarded people usually do not utter complaints of heartburn, it requires a high index of suspicion to discover possible GERD. Therefore it is relevant for care professionals such as nurses to have knowledge of those with a higher risk of GERD and of the possible manifestations of GERD.

**Methods:**

Using a predefined search method, electronic databases were searched for studies relating the presence of symptoms to the presence of GERD. Relevant data were extracted and the methodological quality of the studies assessed. The results of the included studies were synthesized and conclusions about the level of evidence were drawn.

**Results:**

Nineteen studies were found relating symptoms to the presence of GERD. Only four were of good methodological quality. The studies were very diverse concerning the studied population, the study method, and the kind of symptoms examined. This makes it difficult to synthesize the results of the studies. There is evidence that patients with cerebral palsy, patients using anticonvulsive drugs, and those with an IQ lower than 35 more frequently have GERD. There is also evidence that vomiting, rumination and hematemesis are associated with a higher risk of the presence of GERD, whereas there is no clear scientific evidence that particular behavior symptoms are indicative for GERD.

**Conclusion:**

The possible manifestations of GERD are many and varied. A guideline will be made for care professionals to aid systematic observation of possible manifestations of GERD.

## Background

Gastroesophageal reflux disease (GERD) is a very common condition in severely mentally retarded people, with approximately half of the institutionalized population having GERD [1]. GERD occurs when stomach acid frequently backs up (or refluxes) into the gullet (or esophagus). Usually this is felt as heartburn. The consequences of this abnormal acid exposure, if untreated, are serious. It not only reduces the quality of life [2], but can also have serious medical consequences such as inflammation of the inner lining of the gullet (esophagitis), recurrent pneumonia, bronchitis and cancer [3,4]. Therefore it is important that care professionals are sensitive to signals indicative of GERD.

In the general population heartburn is the predominant symptom of GERD[5]. Patients are treated by their physicians to relieve heartburn. There is already a lot of knowledge about the treatment of GERD (the Cochrane Library contains twelve reviews on (aspects of) GERD). Several guidelines for physicians exist for appropriate diagnosis and treatment of GERD in the general population (e.g. from the American College of Gastroenterology [6]). The symptoms mentioned in these guidelines do not usually apply to severely mentally retarded people. For example, they do not utter the usual complaints such as heartburn. Three quarters of those having abnormal concentrations of acid in the gullet (diagnosed by so-called PH measurements), suffered chronic inflammation of the inner lining of the gullet (esophagitis), but did not complain about it [7]. This makes it difficult to detect chronic reflux of acid gastric contents and to assess the effectiveness of a treatment.

Therefore it is important that people offering daily care to mentally retarded people are sensitive to predisposing factors and symptoms of GERD (other than heartburn). These care professionals may provide the physician with valuable information about the behavior of the mentally retarded person. The aim of this review is to find scientific evidence of signals other than heartburn, that can be observed by care professionals (e.g. nurses, therapists) and that imply an increased chance of the presence of GERD. These signals will become the subject of a guideline for care professionals on symptoms and risk factors of GERD. This new guideline will be an extension of an already existing guideline in the Netherlands for physicians working with severely mentally retarded persons [8]. The combination of both guidelines will contribute to a broad multidisciplinary approach to the diagnosis and treatment of GERD in severely mentally retarded people.

### Objectives

Because we did not find a systematic review of studies investigating symptoms of GERD in the population of severely mentally retarded people, we conducted a systematic review to establish the extent to which persons giving daily care to severely mentally retarded people can observe whether that person has a higher risk of having GERD. If there are particular characteristics related to GERD it is important that care professionals know them and can communicate their observations to the physician.

Research questions are:

1. What symptoms are found to be indicative for GERD in severely mentally retarded people?

2. Is there scientific evidence that severely mentally retarded people with GERD behave differently from severely mentally retarded people without GERD?

3. What are found to be predisposing factors of GERD in severely mentally retarded people?

## Methods

### Selection criteria for considering studies for this review

Table 1 shows the criteria for considering studies for this systematic review.

**Table 1:** Selection criteria for including studies

Types of studies:
- empirical studies with a minimal sample size of five subjects
- published from 1990 on.
Types of participants:
- subjects are mentally retarded or have a neurological disorder^1^;
- subjects are at least two years of age.
Types of symptoms:
- all types of observable symptoms that are related to the results of a valid reference test for diagnosing GERD, such as PH-measurement, gastrointestinal endoscopy, radiologic method for the detection of gastroesophageal reflux.

^1 ^People with a serious neurological impairment such as cerebral palsy are often mentally retarded.

### Search strategy

We searched Medline/Pubmed, the Cochrane Database of Systematic Reviews, CINAHL, DARE, Psychinfo, and Embase (date of extraction 27-01-2006). Research terms while searching Medline/Pubmed were (this procedure was adapted to the other databases):

"mentally handicapped" or "mental disability" or "intellectual disability" or "mentally disabled" or "mental retardation" or "intellectually disabled" or "mentally retarded" or "developmental disability" or "neurologically impaired" or "neurologically disabled" or "neurological disability"

and

"gastroesophageal reflux" or GERD or GORD or esophagitis or regurgitate or regurgitation

and

Limits: year of publication: > = 1990

After correction for references found in more than one database, this resulted in 442 titles. We also hand-searched reference lists from (apparently) relevant publications to identify further relevant literature. In addition, some experts gave us a list of references. This finally resulted in 477 potentially relevant references.

Subsequently, titles and abstracts were studied independently by two researchers. All studies that clearly did not meet one of the inclusion criteria were excluded from the review by the first reviewer (AdV). The second reviewer (JB) controlled titles and abstracts of the excluded studies and when she had any doubt about the relevance of the study she reincluded the study. If the studies appeared to meet the inclusion criteria or there was any doubt, the full text was obtained. This resulted in 143 references of which the full text was studied. The two reviewers independently checked if the studies satisfied all criteria in Table 1. Disagreements regarding inclusion status were resolved by discussion. This resulted in 19 publications.

### Analysis of the studies

Subsequently, the two reviewers independently analyzed the methodological characteristics and results of the studies, using the protocol as described below. In the case of different judgments, the reviewers discussed the results in order to reach consensus.

The protocol for data analysis and extraction included the following items:

1. description of the subjects

- number of subjects

- age of the subjects

- severity of the mental retardation

- other diagnoses

- home setting

- intake of food

2. type of symptoms investigated

3. reference test used (PH measurement, gastrointestinal endoscopy, radiologic method)

4. study design

- method of recruiting the subjects (including whether the presence of GERD or GERD symptoms was a criterion for inclusion);

- method of data-collection

5. results of the study and conclusion according to the authors

### Assessment of the level of evidence

The methodological quality of the selected empirical studies was analyzed by the two reviewers (AdV and JB) independently, using a protocol derived from the protocols of the Dutch Cochrane Centre (form I for diagnostic tests). The protocol for rating the quality of the study contained six items for internal validity and two items for external validity. All items were scored as yes, no, or unclear. The two reviewers independently rated the items reflecting the quality of the study and agreed on 78%. If they judged differently they discussed the item until they agreed. If no agreement was reached a third person (AF) was asked for advice (1 item).

Information about five items was not usually found in the publication. Judgment of the validity of the study based on these items was therefore inadequate. Three other items could be answered in the majority of studies, and these items were used to assess the validity of the study. Two items concerning sample size and the uniformity of the procedure to diagnose GERD within one study were added to judge the overall methodological quality of the study. Each item corresponds with 1 quality point. The total number of quality points reflects the judgment of the methodological quality and can vary from 0 to 5 (all quality items were positive).

The items used to judge the methodological quality are:

a. Sample size. Studies with a relatively high number of subjects, i.e. at least the median number of subjects, received an extra quality point.

b. Uniformity of the diagnostic procedure. The sensitivity of diagnostic tests differs. If subjects within one study are diagnosed as GERD- (or non GERD) patients using several diagnostic tools, the reliability of the data may decline. Therefore studies using the same diagnostic procedure for all subjects received 1 point.

c. The (in)dependence of the decision-making procedure with regard to performing a reference test or verification bias. If both the assessment of symptoms and the reference test are not applied to all subjects, verification bias may influence the results. For example, when subjects without symptoms are considered not to have GERD. Or when only subjects with symptoms of GERD such as vomiting are tested with a reference test, because of ethical considerations (i.e. the reference test is considered to be too invasive [9]). When the reference test is not applied to all subjects, the sensitivity of the symptom will be higher, whereas the specificity will be lower (no verification bias = 1 point).

d. Procedure of including subjects in the study or selection bias. If the subjects do not constitute a random sample of the population, selection bias may be present when subjects are selected on certain symptoms (e.g. those who are suspected of being possible GERD patients have a greater chance of being included). Such selection bias means that the sensitivity is likely to be overestimated (no selection bias = 1 point).

e. The resemblance between the subjects in the study and the indicated population (i.e. subjects of at least two years of age and with IQ < 50) or spectrum bias. If the sensitivity is determined in a group representing a higher risk population, sensitivity will be overestimated. Spectrum bias is an aspect of the external validity of the study (no spectrum bias = 1 point).

The two reviewers reached 100% agreement on the total number of quality points for each study.

### Data extraction

In most studies the symptoms that are studied are available as a dichotomy, e.g. the subject vomits daily or not. Therefore if there were sufficient data, 2 (GERD group or control group) × 2 (absence or presence of the characteristic) tables were constructed. Such consistent presentation of the results of each study permits better comparison of the studies.

When we are faced with an individual and need to determine the likelihood that that person has GERD, we are interested in the predictive value of symptoms. Predictive values, however, will vary with the prevalence of GERD in the research group. In the selected studies, different research groups were studied. Prevalence of GERD in the research group varied from 7% [10] to 78% [11]. Because of this, broad range sensitivity or false positive percentage were calculated (predictive values were only mentioned if sensitivity and false positive percentage could not be calculated). Sensitivity of a characteristic is the percentage of all subjects with GERD, diagnosed with the gold standard test, who indeed have that characteristic. In other words: the probability that a characteristic is present in a GERD patient. The false positive percentage is the percentage of all subjects without GERD who have that characteristic (false positive is 100% specificity). Fisher exact tests were used to evaluate the power of the characteristic to discriminate between GERD and non GERD subjects (p < .05).

### Data synthesis

In order to decide on the strength of the evidence that a characteristic predicts the presence of GERD, the studies were added as follows.

1. For each characteristic we calculated the difference between A and B, where:

- A = the sum of the quality points of the studies finding a statistically significant relationship between the presence of GERD and the characteristic;

- B = the sum of the quality points of the studies not finding a statistically significant relationship between the presence of GERD and the characteristic.

By using the quality points as a weight, studies of higher quality have more impact on the results.

We interpreted the difference as:

- A-B > = 3: there is evidence that the characteristic is related to GERD;

- A-B =< -3: there is evidence that no relation exists between the characteristic and GERD;

- -3<A-B<3: the results are contradictory.

If a characteristic was only investigated in one study and this study was of low quality (i.e. 0, 1 or 2 quality points), the characteristic will not be mentioned in the results.

2. A qualification of the evidence was given:

Level A. There is at least one highest quality study (5 quality points), or two independent studies with 4 quality points;

Level B. There are at least two independent studies with 3 quality points, or one study with 4 quality points;

Level C. The studies that do not meet levels A and B.

This synthesis takes into account the outcomes and methodological quality of the studies.

## Results

### Data-extraction and quality assessment

Table 2 contains an overview of the main methodological characteristics of the nineteen included studies. This paragraph describes the setting and data collection, the research group, the reference tests and the way the results may be biased.

**Table 2:** Characteristics of included studies (n = 19)

First author + data collection method+ quality points	Setting	Characteristics research group (number, age, sex, impairment)	Reference test	Verification bias possible?	Selection bias possible?	Spectrum bias possible?
Böhmer et al. [21] Inspection of teeth + reference test q.p. = 5	Random selection of residents with IQ < 50 of 3 institutes for mentally retarded people	GERD group: 28 Control group: 35 (tested negative for GER) Mean age 22.8 years. 48% males, 52% females IQ < 50	24 hour pH test	No	No	No
Böhmer et al. [10] Review of record q.p. = 4	All residents with IQ < 50 of 5 institutes for mentally retarded people	GERD group: 107 Control group: 1,580 (1,518 subjects not tested for GER and 62 subjects tested negative for GER) Mean age 35.3 years (range 2–92 years). 61% males, 39% females IQ < 50	Endoscopy	Yes	No	No
Böhmer et al. [17] Questionnaire + reference test q.p. = 3	Residents of 1 institute for mentally retarded people with IQ < 50 and with symptoms of GER	GERD group: 57 Control group: 46 (tested negative for GER) Mean age 38.4 years (range 4–75 years) 63% males, 37% females IQ < 50	24 hour pH test	No	Yes	Yes
Böhmer et al. [7] Questionnaire + reference test q.p. = 5	Persons with IQ < 50 randomly selected in six also randomly selected institutes for people with intellectual disability.	GERD group: 186 persons Control group: 200 persons (tested negative for GER) Mean age 35 years, 40% females IQ < 50	24 hour pH test	No	No	No
Cameron et al. [23] Review of records q.p. = 2	All subjects without GER who received a gastronomy tube Evaluation of an intervention	Intervention group: 63 children Mean age 3.5 years (range 1 month-17 years) Sex: 52% males, 48% females All children were neurologically impaired	Pretest: UGI-studies, pH test, or esophageal biopsy Post test: ?	Yes	No	Yes
Field et al. [13] Review of records q.p. = 2	All children referred to an interdisciplinary feeding program for the evaluation of feeding problems	GERD group: 178 children Control group: 171 children tested negative or not tested Median age 13–36 months (range 1 month-12 years) 57% boys, 43% girls. 64% with developmental disabilities	Diverse: in most cases endoscopy, pH test and/or radiographic studies	Yes	No	Yes
Gustafsson et al. [11] Review of records + reference test q.p. = 3	All children who visited an outpatient clinic It is not clear for what reasons the children visited the outpatient clinic	GERD group: 25 children Control group: 7 children negatively tested Mean age 11.2 years (range 0.7 – 19 years). 34% boys, 66% girls. All have cerebral palsy with severe brain damage (66%) or severe mental retardation with cerebral atrophy (34%)	24 hours pH test	No	No	Yes
Halpern et al. [19] Review of records and/or questionnaire by parents q.p. = 4	All children referred to a pediatric surgical service for detection, quantification or surgical treatment of GER	GERD group: 69 children Control group: 57 children negatively tested Age range 1 – 16 years. Sex: ? Impairment: 36% CNS disease	18–24 hours pH test	No	No	Yes
Heine et al. [24] Questionnaire + review of records q.p. = 2	All children without or with mild GER after gastronomy Evaluation of an intervention.	Intervention group: 30 children Mean age 6 years (range 3 months-17 years) Sex: 60% boys, 40% girls All children were neurologically impaired 23 had intellectual disability	Pretest: 24 hours pH test + esophageal biopsy Post test: Probably 24 hours pH test + endoscopy	No	No	Yes
Lin et al. [20] Interview (parent) + reference test (to check if the selection was correct) q.p. = 2	All children diagnosed as GERD patients in the University Hospital	GERD group: 34 children. No control group Median age 2.5 years (range 1.1–9.7 years) 53% boys, 47% girls 14 children had a neurological disorder, 10 had other diseases, 10 did not have an underlying disease	24 hour pH test	Yes	No	Yes
Luzzani et al. [12] Questionnaire + reference test q.p. = 2	Patients with Cornelia de Lange Syndrome Strategy of recruitment is unclear	GERD group: 28 Control group: 15 with normal pH test Mean age 6.5 years (range 1 month- 30 years) 18 males and 25 females	24 hour pH test and endoscopy	No	Yes	Yes
Martinez et al. [18] Review of records q.p. = 1	Subjects with antireflux operation and after operation tested with a reference test	GERD group: 47 children Control group: 55 children tested negative Age: children over 2 years of age. Sex: ? All have severe cognitive and motor impairment	Diverse: upper gastrointestinal contrast study (barium), nuclide chalasia scan, upper endoscopy, extended pH study	Yes	Yes	Yes
Orchard et al. [15] Review of records q.p. = 3	All persons who reside in a long-term care facility and are admitted to a hospital with UGI tract bleeding	GERD group: 28 adults Control group: 12 adults Mean age 42 years, 78% males All are mentally retarded, 87% with IQ < 35–40	endoscopy	No	No	Yes
Ravelli et al. [16] Reference test, it is not clear how symptoms were measured q.p. = 2	Children who suffered retching and/or vomiting and were referred to a hospital for suspected gastrointestinal disorder	GERD group: 13 children Control group: 6 children Age: approx. 4.5 years Sex: approx. 50% boys All children selected for this review have CNS disorder and do not suffer gastric dysrhythmias	24 hour pH test and endoscopy	No	Yes	Yes
Rogers et al. [14] Reference test + questionnaire about regurgitation to select subjects q.p. = 2	Subjects living in a residential facility with a history of regurgitating food into the mouth for a minimum of 3 months	GERD group: 16 adults Control group: 7 adults negatively tested Age range 28–65 years 10 males, 13 females All with severe developmental disabilities 61% had cerebral palsy	Radiological studies	No	Yes	No
Shaw et al. [22] Observation of teeth + reference test q.p. = 2	Children attending a dental outpatient clinic of a hospital	GERD group: 12 children. Control group: 9 children with normal pH test Mean age 12.5 years (range 3.9 – 20.7 years) Sex: ? All have cerebral palsy	24 hour pH test	No	Yes	Yes
Wadie et al. [25] Review of records q.p. = 1	All subjects without GER who received a gastronomy tube Evaluation of an intervention.	Intervention group: 56 children Mean age 4.4 years Sex: ? All children were neurologically impaired	Pretest: upper gastrointestinal studies, sometimes followed by a milk scan Post test: ?	Yes	No	Yes
Van Winckel [9] Interview with caregivers + review of records + reference test q.p. = 3	All children of a Pediatric Institute with IQ < 30 combined with severe motor disorders	GERD group: 46 children (45 with symptoms of GERD and tested positive + 1 asymptomatic child that is tested positively) Control group: 38 children (9 with symptoms but negative reference test, 2 without symptoms and negative reference test, 12 without symptoms and not tested, 15 with symptoms but not tested because parents refused the test) Mean age 13 years (range 2–18 years) 52% boys, 48% girls IQ < 30 combined with severe motor disorders	24 hour pH test and endoscopy	Yes	No	Yes
Vega Gutierrez et al. [26] Review of records q.p. = 2	All children with neurological or psychiatric problems who visited an neuropediatric outpatient clinic	GERD group: 27 children Control group: 113 children, not tested or negatively tested Median age 7.7 years (range 8 months-18 years) 67% boys, 33% girls; 32% had moderate to severe mental retardation	Barium ingestion with fluoroscopy and/or eso-phascopy	Yes	No	Yes

? information not found in publication

### Setting and data collection

First we considered how subjects were selected for the study. In eleven studies subjects are not selected on (symptoms of) GERD. In two of these studies, however, the recruitment procedure is not explicitly mentioned in the article and it is therefore not certain whether subjects are not selected on (symptoms of) GERD (see table 2). In the study of Gustafsson et al. [11] the setting is an outpatient clinic and it is not clear why the subjects included visited the clinic. The subjects of the study of Luzzani et al. [12] all had Cornelia de Lange Syndrome but the setting was not clear. The other eight studies included subjects suspected of being GERD patients because of eating problems [13], regurgitation [14], UGI-tract bleeding [15], a range of GERD symptoms [16,17] or already diagnosed as having GERD [18-20].

The data collection methods most often mentioned concerned a retrospective review of (medical) records (11 studies), a questionnaire or interview (8 studies) and reference tests for diagnosing GERD (10 studies). In two studies on the relationship between GERD and erosion of teeth, an inspection of the teeth was conducted for the purpose of the study [21,22].

### Research groups

The number of subjects in each study varies between 19 [16] and 1,687 [10]. The median number of subjects is 63. All but four studies [20,23-25] distinguish a GERD and a non GERD group, allowing a comparison of the symptoms of the GERD and the non GERD groups.

In twelve studies the research group only includes children, and two studies only include adults. In the other five studies both children and adults participate.

In the studies of Böhmer et al. [10,15,17,21], Orchard et al. [15] and Van Winckel [9] only people with severe mental retardation are included. Böhmer et al. [7,10,17,21] include mentally retarded people with IQ < 50. This research group most closely resembles the population of the present systematic review study. Orchard et al. [15] includes mentally retarded adults, most (87%) with IQ < 35/40. However, only when they were admitted to hospital for UGI bleeding was this registered. Van Winckel [9] includes children with IQ < 30 having severe motor disorders. The study of Luzzani et al. [12] only includes children with Cornelia de Lange Syndrome. These children usually have moderate to severe mental retardation, but may also have only mild mental retardation. The other studies do not explicitly include mentally retarded people. They are included in this review because (part of) the research group has a neurological disorder. In these studies information about the level of mental retardation of the research groups is often not provided (see table 2).

### Reference test

In eleven studies the reference test is a pH test. In some studies, most often if the data were only retrospectively gathered by examining the records of patients, there was no uniform way of diagnosing GERD (e.g. Martinez [18]). In these cases contrast studies are more common. Five studies, in which the diagnoses were retrospectively retrieved from the (medical) records, had very diverse reference tests [13,18,23-25]. The test used is likely to influence the results. A less sensitive reference test to diagnose GERD reduces the number of GERD patients and as a result overestimates the sensitivity and underestimates the specificity of a symptom.

### Verification bias

Verification bias might be present when reference testing is conducted only selectively, because the reference test is considered too expensive or too invasive. Also in retrospective studies this might be the case because generally not all subjects were tested for GERD. Only in subjects where GERD was suspected might a test have been done. In eleven studies all subjects were tested with a reference test (table 3). In the other eight studies not all subjects were tested, which may have led to an overestimation of the sensitivity of symptoms.

**Table 3:** Summary of the results of the review

	Difference between GERD and non GERD group			Sensitivity		
	Number of studies	Relation^1^	Level of evidence^2^	Number of studies	Range	median

*GERD symptoms*						
Vomiting	5	yes	A	8	22–100	57
Rumination	3	yes	A	3	31–40	32
Regurgitation	4	no	A	5	29–46	32
Food refusal	3	no	A	4	29–49	38
Hematemesis	6	yes	A	8	4–41	17
Iron deficiency anemia	3	no	A	4	11–35	17
(Recurrent) pneumonia	3	no	A	4	16–52	25
COPD, bronchitis, asthma)	1	no	C	3	11–36	33
Respiratory symptoms	1	no	C	1	-	44
Stridor	1	no	C	1	-	24
Wheezing	1	Yes	C	1	-	43
Failure to thrive	3	+/-^1^	B	5	7–92	40
Adiposity	1	No	B	1	-	18
Dental erosion	2	Yes	A	2	59–75	67
						
*Behavioral symptoms*						
Behavior problems	2	no	A	2	47–48	48
Automutilation	2	no	A	3	19–29	23
Aggression	2	no	A	2	19–23	21
Fear	1	no	A	1	-	11
Episodes of screaming	2	no	A	2	23–24	24
Depression	1	yes	A	1	-	17
Restlessness	2	no	A	2	19–23	21
Pain/irritability	2	+/-	C	3	34–52	44
Heartburn	1	no	C	2	9–12	11
Changed behavior	1	yes	B	1	-	70
						
*Predisposing factors*						
Nonambulance	4	no	A	4	44–58	52
Scoliosis	4	no	A	4	31–50	49
Cerebral Palsy	5	yes	A	5	46–70	61
Use of anticonvulsive therapy	4	yes	A	4	46–72	63
Central Nervous System Disease	1	yes	A	1	-	45
IQ < 35	3	yes	A	3	80–88	82
						
*Other factors*						
Sex (males)	3	no	A	3	56–60	58
Down's syndrome	2	no	A	2	11–13	12
Constipation	4	no	A	4	46–65	60
Melena	1	no	C	1	-	4
Nasogastric feeding	2	no	A	2	4–7	6
Gastrostomy feeding	2	+/-	A	2	11–16	14

^1 ^Statistically significant relation between a characteristic and GERD. +/- indicates that results are contradictory.

^2 ^Level A highest level of evidence, level B = intermediate level of evidence, C = lowest level of evidence.

### Selection bias

The recruitment procedure should be such that all subjects in the population of interest have an equal chance of participating in the research. In six studies selection bias is possible because the method of enrolling people for the study is not clear. In thirteen studies there was probably no (severe) selection bias because subjects were randomly selected [7,21] or because all subjects in the population were included.

Sometimes selection bias might still be present because parents did not give permission to participate or reference tests were not conducted. For example, in the study of Böhmer et al. [7] 15% of the parents gave no permission for pH-monitoring and 11% of the pH tests failed. Also in the study of Van Winckel [9] 16 of the 69 children (23%) were not properly tested or not tested at all because parents gave no permission.

### Spectrum bias

Spectrum bias concerns the question whether the results that are found for the study population are applicable to the indicated population of this review. That is, subjects of at least two years of age with a severe mental disability (IQ < 50). If the population of the study consists of people with a relatively high risk of GERD, the sensitivity and specificity are likely to be overestimated. Only the studies of Böhmer et al. [7,10,17,21] and Rogers et al. [14] have the same population as the indicated population. Böhmer found prevalences for GERD of 7% [10] and 48% [7]. The first study [10] is based on a review of records, whereas in the second study [7] a large group is tested with a reference test, irrespective of the symptoms. This difference in prevalences indicates that GERD might not easily be recognized in the indicated population because there are no observable symptoms (silent GERD) or observable symptoms are not recognized as such.

Also the study population of Rogers et al. [14], concerning adults living in a residential facility for people with severe developmental disability, resembles our population. In a subgroup of people who regurgitate, 70% had GERD.

In the other studies in this review spectrum bias may possibly be present, since the research populations differ from the indicated population of this review. The research populations usually consist of groups with higher risks for GERD. These include people with neurological disorders such as cerebral palsy or people already attending a clinic for feeding problems. The prevalences of GERD in the other studies are all at least 46%. Only Vega Gutierrez et al. [26] reported a lower prevalence of 19% in a group of children with neurological or psychiatric problems visiting an outpatient clinic (with 68% having light or no mental retardation). In the studies with higher- risk research populations, sensitivity and specificity are likely to be overestimated.

Table 2 shows that most studies only have one (2 studies) or two (9 studies) quality points. Four studies received three quality points. Four studies had four or five quality points. Considering the kind of bias in the studies, we conclude that sensitivity and specificity of symptoms are more likely to be overestimated, rather than underestimated. The main reasons for overestimation are the retrospective use of records for extraction of symptoms and/or diagnosis of GERD, the selection of subjects who are tested with a reference test (verification bias), and studies conducted with higher risk populations.

### Symptoms of GERD

Table 3 shows the possible symptoms of GERD that were examined in the studies. The symptoms are categorized as "GERD symptoms" and "behavioral symptoms". Furthermore we looked at "predisposing risk factors" and some other factors. We will only report the results if there are *at least three *studies where the symptom was examined (see column 4 Table 3 for total number of studies). More detailed information about the data extracted from the studies can be found in the appendix [see Additional file 1].

#### GERD symptoms

1. Vomiting. Vomiting was examined in eight studies. For five studies 2 × 2 tables could be reconstructed. Three of them revealed a significant difference in vomiting between the GERD and non GERD group. Two studies did not find a statistically significant relationship between vomiting and the diagnosis of GERD. Sensitivity in the eight studies ranged from 22% to 100% (median 57%). Vomiting in the non GERD group was less frequent (5 studies, range 13%–31%, median 14%).

2. Rumination (bringing back up and re-chewing partially digested food that has already been swallowed). Two out of three studies investigating the correlation between rumination and GERD, found that GERD patients more often ruminate than people without GERD [7,10]. Another study [17] found no relation between rumination and GERD.

3. Regurgitation. Six studies examined regurgitation. In one study only a positive predictive value (PPV) was calculated: 70% of the subjects who regurgitate were diagnosed as GERD patients [14]. The authors claim that the PPV is even higher when a more sensitive reference test was used. In the other five studies sensitivity ranged from 29% to 36%. However, no statistically significant differences were found between the GERD and non GERD group.

4. Food refusal. Four studies investigated the relationship between the presence of GERD and food refusal. Although food refusal often occurs, it is not found to be related to the presence of GERD.

5. Hematemesis (vomiting of blood). Nine studies examined hematemesis as a symptom of GERD. Most often the presence of hematemesis in the GERD group differs from the presence in the non GERD group. Sensitivity in the GERD group ranges from 4% to 41% (median 18%). Only a few people diagnosed as not having GERD have hematemesis (range 0%–14%, median 6%).

6. Iron deficiency anemia was examined in four studies, with a sensitivity ranging from 11% to 35%. In one study there was a significant difference between anemia in the GERD and non GERD groups [10]. Two other studies did not find a difference [7,17].

7. Pneumonia and other respiratory symptoms. No relationship was found between (recurrent) pneumonia and GERD in the three studies focusing on this relationship. Respiratory symptoms are examined in several studies. However, the definition varies widely. The overall view shows that such symptoms are not specific for GERD.

8. Failure to thrive. Five studies examined the relation between GERD and failure to thrive, with a sensitivity range of 7%–92%. 2 × 2 tables could be reconstructed in three studies. Results were contradictory. In one study the GERD group significantly differed from the non GERD group [10], whereas in the other studies this was not the case [16,18].

#### Behavioral symptoms

Nine studies also looked at behavioral symptoms of GERD. Because of the wide range of behaviors studied, the results are difficult to compare. Even within one category of behavior, the definitions between studies vary. The overall picture is that behavioral problems are not indicative for GERD. Two behaviors were the subject of research in at least three studies:

1. Automutilation. From these studies we conclude that automutilation is not a symptom that is specific to GERD.

2. Pain/irritability. For pain or irritability the results are not univocal. Van Winckel [9] found a relationship between irritability and GERD. Irritability is defined as inconsolably crying or irresolvable discomfort. The crying or tension cannot be stopped. Martinez et al. [18] found no relation between "pain/irritability" (not further described) and GERD. Vega Gutierrez et al. [26] found "irritability and/or crying" present in 44% of the children with GERD.

#### Predisposing factors

1. Nonambulance. In four studies the relation between nonambulance and GERD was studied. In all studies the GERD group had a somewhat higher percentage of nonambulant people than the non GERD group. However, in only one study did the difference reach statistical significance.

2. Scoliosis. The same studies also investigated the relationship between scoliosis and GERD. As with nonambulance, the GERD group had a higher percentage of people with scoliosis than the non GERD group but this only reached statistical significance in one study.

3. Cerebral palsy. Four out of five studies investigating the relationship between cerebral palsy and GERD concluded that people with cerebral palsy have higher risks of suffering from GERD. Sensitivity ranges from 46% to 70%. In the groups without GERD, the percentages of cerebral palsy patients were significantly lower, ranging from 2% to 41%. In one study only the positive predictive value was presented [22]: 57% of the children with cerebral palsy were GERD patients.

4. Use of anticonvulsive therapy. Four studies examined the use of anticonvulsive therapy and one studied "central nervous system disease", often seizure disorder (42%). All studies found a significant difference in the use of anticonvulsive drugs between the GERD and the non-GERD group. Sensitivity ranges from 45% to 72%, whereas the percentage in the non GERD group ranges from 25% to 53%.

5. Severity of mental retardation. Four studies examined the relation between severity of the mental retardation and GERD. In three studies a difference was found: subjects with an IQ < 35 more frequently had GERD than people with an IQ of between 35 and 50 [7,10]. Vega Gutierrez et al. [26] found that children with moderate to severe mental retardation more often had GERD than children with light or no mental retardation. In another study of Böhmer [17] this relation was not found.

#### Other factors

1. Sex. No relationship has been found between sex and GERD.

2. Constipation. Three out of four studies found no difference between constipation in GERD patients and people without GERD.

3. Gastrostomy feeding. Two studies compared the presence of gastrostomy feeding in people with and without GERD [7,10]. In one study the GERD patients more frequently had gastrostomy feeding [10]. In the other study no differences were found [7]. Three studies were included that evaluated the presence of GERD after placement of a gastrostomy tube [23-25]. In these studies the subjects were not diagnosed as GERD patients before placement. No further antireflux surgery was done (e.g. Nissen fundoplication). The percentage of subjects who developed GERD afterwards was 60%, 27% and 6% respectively. It can be concluded that gastrostomy feeding may trigger GERD, but the evidence is not firm.

## Discussion

The systematic review as described has some limitations. In the first place our research question concerns the population of severely mentally disabled people, and only the studies of Böhmer [7,10,17,21] are found to specifically focus on this population. Many studies focus on people with neurological disorders without mentioning whether they are mentally retarded. It remains unclear whether the neurological disorder or the low IQ is related to GERD. Van Winckel [9] studied persons with a low IQ (< 30) *and *severe neuromotor disorders. She compared the presence of symptoms in persons who could not sit or could not move autonomously with persons who could move by themselves. The latter group had only minor symptoms of GERD. Van Winckel concluded that GERD is primarily due to a neurological disorder. Böhmer et al. [7,10], however, also found a relationship with IQ. These differences indicate that different study populations may lead to different conclusions.

Another point relates to the kind of symptoms the studies focus on. The total number of different symptoms and factors that were studied was about 50 [see Additional file 1]. This broad range of symptoms makes it difficult to pool them and to draw conclusions. And even within one single symptom or factor, operationalization differs between studies. For example vomiting is defined as daily vomiting, including regurgitating [9]; as persistent vomiting and regurgitation being two separate symptoms [7]; or as repeated regurgitation (without mentioning vomiting as another symptom) [11]. Most studies do not give exact definitions of the symptoms, making clustering of results difficult.

Thirdly, in most studies no multivariate analyses were performed to search for clusters of symptoms that distinguish the GERD patients from non GERD patients.

The fourth limitation concerns the way the subjects of the studies were recruited. In eight studies the subjects were selected for the study because they already had possible symptoms of GERD. Therefore it was not possible to detect persons who have GERD without exhibiting symptoms of GERD.

## Conclusion

GERD in mentally disabled individuals is documented frequently and may have serious consequences for the quality of life. Therefore it is very important that GERD is diagnosed and treated properly. In the introduction to this article we stated that severely mentally retarded people with GERD often do not utter complaints of heartburn, and that it is important that professionals giving daily care to severely mentally retarded people observe symptoms of possible GERD and communicate their observations to a physician. From this review it can be conclused that vomiting, rumination and hematemesis are associated with a higher risk of the presence of GERD. There is no clear evidence that particular behavioral symptoms are indicative for GERD. Patients with cerebral palsy, patients using anticonvulsive drugs, and those with an IQ lower than 35 more frequently have GERD.

What do the results of this systematic review imply for the role of nurses and other care professionals in observing symptoms of GERD? First, care professionals should know that all severely mentally retarded people are at risk of having GERD, with approximately half of the institutionalized population being diagnosed as GERD patients [1]. Within this population the risk is even higher in persons with an IQ lower than 35, persons with cerebral palsy, and those using anticonvulsive drugs.

Second, a person who vomits blood or frequently vomits (without blood) has a higher risk of having GERD. Also if a person ruminates, i.e. brings back up and re-chews partially digested food that has already been swallowed, there is a higher risk of GERD.

Third, the first two points must be regarded with caution and are only indicative. Until now the number of studies of sufficient scientific quality on the symptoms of GERD is limited. As a consequence no clearly related symptoms can help care professionals to detect (possible) GERD. The studies of Böhmer et al. [7,10,21] are the only high quality studies found concerning symptoms of GERD, as they examine GERD prospectively in an randomly selected population of severely mentally retarded people.

Fourth, since there is no single symptom that is clearly related to GERD, those giving care to severely mentally handicapped people should be alert to a broad range of symptoms. Böhmer et al. [7] found that in subjects without previous evidence of serious GERD (esophagitis diagnosed by endoscopy), the combination of an IQ < 35 or cerebral palsy plus scoliosis, the use of anticonvulsant drugs, hematemesis, rumination, and depression was predictive for GERD in 68% of the cases without a history of GERD in the past, with a sensitivity of 78% and a specificity of 59%.

Finally, although care professionals do have an important role in observing the condition of severely mentally retarded people, it is of course the physician who diagnoses and treats GERD. Good medical treatment of GERD is of importance in the population of severely mentally disabled people, with nearly 50% of those with IQ < 50 having GERD [7]. The lack of a strong association between symptoms and GERD stresses the important role of physicians. Therefore physicians with adequate knowledge of diagnosing and treating GERD in this population should always be involved in the care process. Care professionals can make a valuable contribution by closely observing the person they care for and by communicating their observations to the physician. The results of this review are used to write a guideline about GERD for professionals giving care to severely mentally retarded people. Together with the already existing guideline for physicians, it will contribute to better care and better quality of life for the severely mentally retarded.

## Competing interests

The authors declare that they have no competing interests.

## Authors' contributions

AJEdV carried out the study and drafted the manuscript. JTB carried out the study and drafted the manuscript. RCNdB participated in the design of this study (criteria to select relevant studies and to rate the studies) and commented drafts of the manuscript. CJMB participated in the design of this study (criteria to select relevant studies and to rate the studies) and commented drafts of the manuscript. ALF contributed to the conception, design, analysis and interpretation of data, critically commented on the several steps in the study and the earlier drafts of this manuscript. All authors agreed on this manuscript

## Pre-publication history

The pre-publication history for this paper can be accessed here:



## Supplementary Material

Additional file 1 Appendix with the results of the studies included in the review. Click here for file
