# Supplementary material for: Symptoms of gastroesophageal reflux disease in severely mentally retarded people: a systematic review
Source: BMC Gastroenterol. 2008 Jun 11;8:23. doi: 10.1186/1471-230X-8-23 (PMC2435531; doi:10.1186/1471-230X-8-23)
Supplement: Additional file 1 — Appendix with the results of the studies included in the review. [file 1471-230X-8-23-S1.doc]

Appendix. Results of the studies included in the review (the number of quality points is mentioned after each reference)

| Symptom | Sensitivity-False positive percentage^1^  Fisher exact p<.05 | Sensitivity-False positive percentage^1^  Fisher exact p>=.05 | Remarks |
| --- | --- | --- | --- |
| *GERD symptoms* |  |  |  |
| Vomiting | 22%-13% (Böhmer et al. [7] (5))  60%-31% (Martinez et al. [18] (1))  84%-14% (Böhmer et al. [10] (4)) | 30%-13% (van Winckel [9] (3))  39%-24% (Böhmer et al. [17] (3)) | Lin et al. [20] (2): sens=100%  Luzzani et al. [12] (2): sens 54%  Vega Gutierrez et al. [26] (2): vomiting or regurgitation sens=74% |
| Rumination | 31%-14% (Böhmer et al. [7] (5))  40%-10% (Böhmer et al. [10] (4)) | 32%-33% (Böhmer et al. [17] (3)) |  |
| Regurgitation |  | 29%-21% (Böhmer et al. [7] (5))  32%-14% (Gustafsson et al. [11] (3))  36%-29% (Böhmer et al. [10] (4))  46%-33% (Böhmer et al. [17] (3)) | Rogers et al. [14] (2): PPV^2^=70%  Luzzani et al. [12] (2): sens: 32% |
| Food refusal | 46%-20% (Field et al. [13] (2)) | 29%-22% (Böhmer et al. [7] (5))  49%-33% (Böhmer et al. [17] (3)) | Van Winckel [9] (3): also included in this study. Because of missing data, a 2x2 table could not be reconstructed. Conclusion of the Author: no relationship. PPV=50%  Luzzani et al. [12] (2): sens 29% |
| Hematemesis | 14%-3% (Böhmer et al. [7] (5))  32%-5% (Martinez et al. [18] (1))  41%-0% (van Winckel [9] (3))  32%-9% (Böhmer et al. [10] (4)) | 12%-14% (Gustafsson et al. [11] (3))  4%-7% (Böhmer et al. [17] (3)) | Martinez et al. [18] (1): the authors use the term “gastrointestinal bleeding” as a symptom. They do not further specify how this is observed.  Orchard et al. [15] (3): “UGI tract bleeding, defined as hematemesis, coffee ground emesis, or melena , with positive results of a Stool Hemoccult test” PPV=70%  Lin et al. [20] (2): sens=15%  Vega Gutierrez et al. [26] (2): sens=19% |
| Iron deficiency anemia | 35%-18% (Böhmer et al. [10] (4)) | 15%-12% (Böhmer et al. [7] (5))  11%-11% (Böhmer et al. [17] (3)) | Vega Gutierrez et al. [26] (2): sens=19% |
| (Recurrent) pneumonia |  | 25%-19% (Böhmer et al. [7] (5))  52%-41% (Martinez et al. [18] (1))  16%-17% (Böhmer et al. [17] (3)) | Lin et al. [20] (2): sens=24% |
| Chronic obstructive pulmonary diseases, bronchitis, asthma |  | 11%-13% (Böhmer et al. [17] (3)) | Vega Gutierrez et al. [26] (2): sens=33%  Luzzani et al. [12] (2): infection of respiratory tract sens 36% |
| Cough |  |  | Luzzani et al. [12] (2):sens 11%  Lin et al. [20] (2): nocturnal cough sens=9% |
| Apnea |  | 30%-25% (Martinez et al. [18] (1)) |  |
| Respiratory other than pneumonia (difficult breathing, wheezing, cyanosis, asthma, coughing) |  | 45%-49% (Martinez et al. [18] (1)) |  |
| Respiratory symptoms |  | 44%-71% (Gustafsson et al. [11] (3)) |  |
| Selectivity of food by texture | 21%-32% (Field et al. [13] (2)) |  | Field et al. [13] (2): the GERD group shows less selectivity than the group with eating problems without GERD |
| Selectivity of food by type | 16%-27% (Field et al. [13] (2)) |  | Field et al. [13] (2): the GERD group shows less selectivity than the group with eating problems without GERD |
| Choking |  | 38%-53% (Martinez et al. [18] (1)) | Lin et al. [20] (2): sens=24%  Martinez et al. [18] (1) “choking-gagging-retching” |
| Failure to thrive or malnutrition | 36%-21% (Böhmer et al. [10] (4)) | 21%-9% (Martinez et al. [18] (1))  92%-67% (Ravelli et al. [16] (2)) | Lin et al. [20] (2): sens=44%  Vega Gutierrez et al. [26] (2): sens=7% |
| Adiposity |  | 18%-7% (Böhmer et al. [10] (4)) |  |
| Hoarseness |  |  | Lin et al. [20] (2): sens=3% |
| Stridor |  | 24%--36% (van Winckel [9] (3)) |  |
| Wheezing | 43%-13% (van Winckel [9] (3)) |  |  |
| Dental erosions | 59%-32% (Böhmer et al. [21] (5)) | 75%-33% (Shaw et al. [22] (2)) |  |
|  |  |  |  |
| Behavioral symptoms |  |  |  |
| Behavior problems |  | 48%-47% (Böhmer et al. [7] (5))  47%-44% (Böhmer et al. [17] (3)) |  |
| Automutilation |  | 19%-20% (Böhmer et al. [7] (5))  29%-7% (Luzzani et al. [12] (2))  23%-26% (Böhmer et al. [17] (3)) |  |
| Aggression |  | 19%-20% (Böhmer et al. [7] (5))  23%-17% (Böhmer et al. [17] (3)) |  |
| Fear |  | 11%-7% (Böhmer et al. [7] (5)) |  |
| Episodes of screaming |  | 24%-26% (Böhmer et al. [7] (5))  23%-15% (Böhmer et al. [17] (3)) |  |
| Depression | 17%-8% (Böhmer et al. [7] (5)) |  |  |
| Restlessness |  | 19%-14% (Böhmer et al. [7] (5))  23%-11% (Böhmer et al. [17] (3)) |  |
| Hyperactivity | 86%-13% (Luzzani et al. [12] (2)) |  |  |
| Nocturnal agitation | 50%-7% (Luzzani et al. [12] (2)) |  |  |
| Bruxism |  | 36%-7% (Luzzani et al. [12] (2)) |  |
| Pain/irritability | 52%-13% (van Winckel [9] (3)) | 34%-35% (Martinez et al. [18] (1)) | Vega Gutierrez et al. [26] (2): sens=44% |
| Heartburn |  | 12%-14% (Gustafsson et al. [11] (3)) | Lin et al. [20] (2): sens=9% |
| Changed behavior | 70%-54% (Böhmer et al. [10] (4)) |  | Böhmer et al. [10] (4): in the original article the sensitivity is 40%, indicating that the GERD group shows less changed behavior. In another publication about the same study [1], the sensitivity is 70%. This is probably the correct percentage because the authors conclude that “our study demonstrates that behavior problems are symptoms indicating reflux oesophagitis..”. |
|  |  |  |  |
| Predisposing risk factors |  |  |  |
| Nonambulance | 57%-47% (Böhmer et al. [7] (5)) | 44%-38% (Böhmer et al. [10] (4))  58%-50% (Böhmer et al. [17] (3))  46%-33% (Ravelli et al. [16] (2)) |  |
| Scoliosis | 50%-28% (Böhmer et al. [7] (5)) | 48%-40% (Böhmer et al. [10] (4))  49%-35% (Böhmer et al. [17] (3))  31%-17% (Ravelli et al. [16] (2)) |  |
| Cerebral palsy  (including neurological syndromes such as hemi-, tetra-, quadriplegia and overall hypotonia) | 61%-35% (Böhmer et al. [7] (5))  70%-2% (Vega Gutierrez et al. [26] (2))  49%-38% (Böhmer et al. [10] (4))  63%-41% (Böhmer et al. [17] (3)) | 46%-33% (Ravelli et al. [16] (2)) | Vega Gutierrez et al. [26] (2): only tetraparetic cerebral palsy  Ravelli et al. [16] (2): “spasticity”  Shaw et al. [22] (2): PPV=57% |
| Central Nervous System Disease | 45%-25% (Halpern et al. [19] (4)) |  | Halpern et al. [19] (4): Most often mental-motor retardation and/or seizure disorder |
| Use of anticonvulsive therapy (including all benzodiazepines) | 72%-53% (Böhmer et al. [7] (5))  59%-29% (Böhmer et al. [10] (4))  67%-44% (Böhmer et al. [17] (3)) |  |  |
| Seizures |  | 46%-33% (Ravelli et al. [16] (2)) |  |
| IQ<35 | 82%-69% (Böhmer et al. [7] (5))  80%-65% (Böhmer et al. [10] (4)) | 88%-85% (Böhmer et al. [17] (3)) |  |
| Moderate to severe mental retardation (not having tetraparetic cerebral palsy) | 93%-18% (Vega Gutierrez et al. [26] (2)) |  |  |
| Hyperextensive posturing |  | 13%-16% (Martinez et al. [18] (1)) |  |
| Oral motor delays (e.g. problems with chewing, tongue movement, lip closure) |  | 43%-46% (Field et al. [13] (2)) |  |
| Neurological symptoms (e.g. spasticity, Sandifer´s syndrome) |  |  | Vega Gutierrez et al. [26] (2): sens: 41% |
|  |  |  |  |
| Other contributing factors |  |  |  |
| Sex (males) |  | 58%-61% (Böhmer et al. [7] (5))  60%-57% (Böhmer et al. [10] (4))  56%-72% (Böhmer et al. [17] (3)) |  |
| Down’s syndrome |  | 11%-10% (Böhmer et al. [7] (5))  13%-7% (Böhmer et al. [17] (3)) |  |
| Constipation | 64%-33% (Böhmer et al. [10] (4)) | 65%-55% (Böhmer et al. [7] (5))  46%-33% (Ravelli et al. [16] (2))  56%-57% (Böhmer et al. [17] (3)) |  |
| Melena (zwarte stoelgang) |  | 4%-14% (Gustafsson et al. [11] (3)) |  |
| Nasogastric feeding |  | 7%- 6% (Böhmer et al. [7] (5))  4%-6% (Böhmer et al. [10] (4)) |  |
| Gastrostomy feeding | 16%-4% (Böhmer et al. [10] (4)) | 11%-10% (Böhmer et al. [7] (5)) | 6% of the children who did not have GERD before gastrostomy tube placement, developed GERD afterwards (Wadie et al.[25] (1))  27% of the children who did not have GERD or mild GERD before gastrostomy tube placement, developed GERD afterwards (Heine et al. [24] (2))  60% of the children not treated for GERD required medical treatment or fundoplication for GERD afterwards (Cameron et al. [23] (2)) |
| Assisted feeding |  |  |  |
| Dysphagia |  | 25%-20% (Field et al. [13] (2)) | Lin et al. [20] (2): sens=9% |

1. false positive percentage=100%-specificity
2. PPV= positive predictive value=percentage of persons with a symptom who test positive as GERD patients
